# *Snakin-1* affects reactive oxygen species and ascorbic acid levels and hormone balance in potato

**DOI:** 10.1371/journal.pone.0214165

**Published:** 2019-03-25

**Authors:** Vanesa Nahirñak, Máximo Rivarola, Natalia Inés Almasia, María Pilar Barrios Barón, Horacio Esteban Hopp, Denis Vile, Norma Paniego, Cecilia Vazquez Rovere

**Affiliations:** 1 Instituto de Biotecnología, CICVyA, CNIA, Instituto Nacional de Tecnología Agropecuaria (INTA), Buenos Aires, Argentina; 2 Consejo Nacional de Investigaciones Científicas y Técnicas (CONICET), Buenos Aires, Argentina; 3 LEPSE, Univ Montpellier, INRA, SupAgro, Montpellier, France; 4 INTA LABINTEX Agropolis International, Montpellier, France; Universidade Federal de Vicosa, BRAZIL

## Abstract

*Snakin-1* is a member of the *Solanum tuberosum* Snakin/GASA family. We previously demonstrated that *Snakin-1* is involved in plant defense to pathogens as well as in plant growth and development, but its mechanism of action has not been completely elucidated yet. Here, we showed that leaves of *Snakin-1* silenced potato transgenic plants exhibited increased levels of reactive oxygen species and significantly reduced content of ascorbic acid. Furthermore, *Snakin-1* silencing enhanced salicylic acid content in accordance with an increased expression of SA-inducible *PRs* genes. Interestingly, gibberellic acid levels were also enhanced and transcriptome analysis revealed that a large number of genes related to sterol biosynthesis were downregulated in these silenced lines. Moreover, we demonstrated that Snakin-1 directly interacts with StDIM/DWF1, an enzyme involved in plant sterols biosynthesis. Additionally, the analysis of the expression pattern of PStSN1::GUS in potato showed that *Snakin-1* is present mainly in young tissues associated with active growth and cell division zones. Our comprehensive analysis of *Snakin-1* silenced lines demonstrated for the first time in potato that *Snakin-1* plays a role in redox balance and participates in a complex crosstalk among different hormones.

## Introduction

Reactive oxygen species (ROS) participate in signaling in response to biotic and abiotic stresses as well as in plant development [1, 2]. ROS have been involved in different processes such as root hair and pollen tube growth, stomatal movements and plant–microbe interactions [3–8]. This signaling role implies that ROS homeostasis needs to be tightly controlled within plant cells [2, 9, 10]. Ascorbic acid is a major antioxidant that ensures protection of plant cells against ROS generated by physiological processes as well as by stresses [11, 12]. It has multiple functions in metabolism, electron transport, plant responses to pathogens and abiotic stress and it is also considered to influence plant growth and development through its effects on the cell cycle and cell elongation [13, 14].

Plant growth and development require the integration of many external and internal signals that, together with the intrinsic genetic program, determine plant form and function. Plant hormones are growth regulators that fulfill essential functions during this process [15]. They coordinate endogenous developmental processes and also transform different stimuli to perform adaptive responses to biotic and abiotic stresses [16]. Notably, all of them can modulate various processes single-handedly and independently. Previous investigations have significantly improved our knowledge on how a single hormone can affect plant growth, development and stress responses [17]. However, over recent years, it has become evident that the result of each hormone effect is determined by the crosstalk between several hormones. In fact, hormonal pathways are interconnected through a complex network of interactions and feedback regulations [16, 18]. Interestingly, ROS production is part of the mechanism of many hormones to modulate plant growth and development but the specific roles of these molecules in hormonal signaling pathways are not completely understood yet [19].

Snakin/GASA peptides have been shown to participate in plant growth and development as well as in plant responses to biotic and abiotic stresses [20]. Even though many of them were characterized and involved in different biological functions, their mechanism of action is not completely elucidated. Snakin/GASA proteins are characterized by three domains: a putative signal peptide; a variable region (which differs in its sequence and number of aminoacids among different family members); and a C-terminal region of approximately 60 aminoacids, named GASA domain, with 12 cysteine residues [21]. The conserved position of these cysteines of all Snakin/GASA peptides suggests that they play an essential role. Wigoda et al. (2006) [22] proposed that Snakin/GASA proteins are implicated in redox regulation given that they have putative redox-active sites (i.e. pairs of cysteines separated by one or two aminoacids). Several studies support this hypothesis: the expression of *GIP2*, *GIP4* and *GIP5* in petunia as well as *FaGAST2* in strawberry is induced by H_2_O_2_. Also, the overexpression of some Snakin/GASA genes suppresses the accumulation of ROS in Arabidopsis and petunia [22–24]. In addition, mutation of some of the cysteines of the GASA domain reduces the redox activity of Arabidopsis GASA4 and GASA5; which suggests that these residues are fundamental for their activity as antioxidants [23, 25].

On the other hand, the expression of most of Snakin/GASA genes is modulated by plant hormones [20]. Furthermore, some Snakin/GASA genes play a role in hormonal signaling pathways through the regulation of hormonal responses and levels. For example, Wang et al. (2009) [26] demonstrated that rice OsGSR1 modulates brassinoesteroids (BRs) levels by its direct interaction with a BR biosynthetic enzyme and that it also modulates the gibberellic acid (GA) response by downregulating the expression of the DELLA protein. Additionally, the overexpression of the GA-upregulated *FsGASA4* enhances the content of salicylic acid (SA) and also the expression of genes related to its biosynthesis and responses [27]. Finally, SA signaling is blocked in the *GASA5*-overexpressing plants and this blockage is partially restored by treatment with GA [28].

*Snakin-1* is the first member of the Snakin/GASA family isolated from *Solanum tuberosum* that was shown to have antimicrobial activity *in vitro* [29]. We demonstrated that its overexpression in potato improves resistance to commercially important pathogens; which suggests that it also has *in vivo* antimicrobial activity [30]. Recently, we have shown that *Snakin-1* plays a role in plant growth and development, besides having a function in defense, since its silencing results in altered cell division, leaf primary metabolism and cell wall components [31]. Moreover, we demonstrated that the expression of *Snakin-1* is negatively regulated by GA and is affected by bacterial and/or fungal inoculation in *S*. *tuberosum* cv Kennebec [32].

In the present work, we performed an integrated study of *Snakin-1* silenced potato plants demonstrating for the first time that *Snakin-1* plays a role in redox balance and participates in a complex crosstalk among different hormones.

## Materials and methods

### Plant materials

Silenced (A2 and A3) *Snakin-1* potato lines [31] and wild type (WT) plants were maintained *in vitro* by periodic micropropagation in growth chambers (CMP 3244; Conviron, Manitoba, Canada) at 18 to 22°C, under an 8/16-h dark/light cycle. For the analyses presented in this study, plants were transferred to soil and grown in the greenhouse at 18–25°C under a 10/14-h dark/light cycle and samples of 8-week-old plants were collected. For time-course PStSN1::GUS expression analysis *in vitro* plants were used.

### ROS accumulation in potato leaves

3,3′-Diaminobenzidine (DAB) histochemical staining assays were performed as follows: leaf discs were collected and placed in a solution of DAB-HCl (Sigma-Aldrich) (1 mg.ml^-1^) pH 3.8, as previously described [33]. Peroxide level was also analyzed by the oxidation of 2′,7′-Dichlorodihydrofluorescein diacetate (H_2_DCF-DA) (Sigma-Aldrich), as described by Ezaki *et al*. (2000) [34]. Potato leaves were incubated 10 min in 50 μM H_2_DCF-DA (10 mM Tris-HCl pH 7.2 and 50 mM KCl). Subsequently, they were washed and scanned with a Typhoon 9400 (GeHealthcare). Detection of peroxides production over time was determined with H_2_DCF-DA by incubating leaf discs with the solution and measuring changes in absorbance with a spectrophotometer (488 nm) [35].

For nitrotetrazolium blue chloride (NBT) staining, leaf discs were submerged in an NBT 0.1% solution in 50 mM potassium phosphate buffer, pH 7.8, as previously described by Wohlgemuth et al. (2002) [36].

For DAB and NBT staining, solutions were infiltrated into leaf tissues by 2-min vacuum shocks in a vacuum chamber. Then, the infiltrated leaf tissues were incubated overnight (DAB) or for 2 h (NBT). Leaves were cleared in boiling ethanol (96%, vol/vol) to remove chlorophyll. H_2_O_2_ was visible as a brown precipitate in the tissue and superoxide anion was detected as a blue formazan precipitate.

The experiments were repeated three times. Quantification of the staining (NBT and DAB staining) was performed with Image J (http://rsb.info.nih.gov/ij/) in arbitrary units (mean ± SD).

### Ascorbic acid measurement

100 mg of leaf tissue was homogenized in 1 ml of 3% (v/v) trifluoracetic acid (TFA). The supernatant (500 μl) was injected to an Extract-clean C18 column (Alltech Associates, USA) equilibrated with 100 mM phosphate buffer (pH 7) and eluted with 1.5 ml of phosphate buffer (pH 7). 500 μl of 100 mM K_2_HPO_4_ buffer (pH 8.5) was added to 500 μl of the eluate. To quantify the total ascorbic acid, 15 μl of 0.1 M dithiothreitol and 125 μl of 3% (v/v) TFA were added to 500 μl of extraction mix. To measure the reduced ascorbic acid, 15 μl of 100 mM phosphate buffer (pH 7) and 125 μl of 3% (v/v) TFA were added to 500 μl of the extraction mix. Both samples were incubated for 3 min at room temperature. Ascorbic acid detection was achieved by injecting samples onto a silica-based, reversed- phase C18 column (particle size 5 pm, 150 × 4.6 mm, HL90-5s, Bio-Sil; Bio-Rad, Munich). The mobile phase consisted of a KH_2_P04 buffer (100 mM) at pH 3.0 (with phosphoric acid) and was delivered isocratically at a flow rate of 0.5 ml/min. Ascorbic acid resulted in a peak at 3.5 min. Total ascorbic acid (reduced plus oxidized) concentration was determined after reduction with dithiothreitol. The amount of oxidized DHA was then estimated as the difference in peak area between unreduced and reduced samples.

### GA and SA determination

GA and SA were extracted from lyophilized leaf tissues according to Rosello *et al*., 2016 [37] and Conti *et al*., 2012 [38] respectively. Briefly, a total of 50 ng of [2H4]-SA and 100 ng of [2H2]-GA_1_ were added as internal standards. HPLC was performed using a Waters Alliance 2690 system (Milford, MA, U.S.A.). Aliquots were injected on a Nucleosil ODS reversed-phase column C18 (100 by 2.1 mm, 3 μm). The identification and quantification of hormones were performed by quadruple tandem mass spectrometer (Quattro Ultima, 196 Micromass, Manchester, UK) fitted with an electrospray ion (ESI) source, in multiple reactions monitoring mode (MRM). Precursor ions and their transitions were used as indicated: to SA, 137>93 and its internal standard 215>59 and to GA1, 347>273 and its internal standard 349>275. The spectrometry software used was MassLynx V. 4.1 202 (Micromass).

### RNAseq analysis

For the RNA-sequencing experiment, *in vitro* micropropagated plants were transferred to soil and grown in the greenhouse cycle. We used the *Snakin-1* silenced line A2 and WT for our RNA sequencing study. Young leaves from six independent plants were harvested and total RNA was isolated using RNeasy Plant kit (Qiagen). Samples were pooled from three plants to form one replicate sample (we used two pooled biological replicates per line).

The quality and concentrations of the libraries was determined using a Bioanalyzer (Agilent) and sequenced on the SOLID platform. The reads were obtained in raw color-spaced format. Because of the nature of this format, we used color-space capable algorithms to align the raw reads. First we tried tophat with the–C (color-space) parameter, which gave very poor alignment (~15% mapped). Subsequently, we ran the NovoAlignCS with quality recalibration (-k parameter) and polyclonal filter (-p) (www.novocraft.com/documentation/novoaligncs-3) mapper, which performed much better; on average 60% of reads were mapped. In both cases we used the potato reference genome sequence (PGSC_DM_v3.4_gene.fasta.zip, http://solanaceae.plantbiology.msu.edu/pgsc_download.shtml). After aligning with NovoAlignCS, we used the resulting “bam” aligned files to check for the quality of reads using FastQC (http://www.bioinformatics.bbsrc.ac.uk/projects/fastqc/). This quality check is more robust than color space raw reads because it takes into consideration the effect of quality recalibration and is not so biased by one miscall, which would throw off all latter bases in read, due to the way color-space sequencing works. We observed the typical pattern of solid color-space quality values and all other metrics including GC, or base composition per read or position to be correct. The reads that aligned to the genome were quantified by the Cufflinks programs [39], which provided relative abundance values by calculating fragments per kilobase of exon per million fragments mapped (FPKM) [40, 41]. Cufflinks was also used to find isoforms, promoters, translation start sites and sites of alternative splicing. We also used Cuffmerge to find novel genes or isoforms given our data. The differential expression analysis of all genes was performed using Cuffdiff package-2.2.1 [39] normalizing counts with the upper-quartile method. The Cuffdiff results were compiled and visualized using the R package CummeRbund, Version 2.0 (http://bioconductor.org/packages/release/bioc/html/cummeRbund.html).

### qRT-PCR

RNA from young and fully expanded leaves was isolated using RNAquous kit (Ambion). For qRT-PCR, RNA was treated with DNase (Invitrogen). Reverse transcription reactions were carried out with SuperScript III (Invitrogen) and random primers. All qRT-PCR were performed by an ABI PRISM 7500 (AppliedBiosystems) with SYBR Green master mix (Qiagen). Three biological and technical replicates for each gene were run and qRT-PCR data analyses and primer efficiencies were obtained with LinRegPCR software [42]. Potato elongation factor 1α (Ef1α) was used as an internal control [43]. All primers used in this study are listed in S1 Table.

### Bimolecular fluorescence complementation (BiFC) analyses

The coding sequence of *Snakin-1* (with or without its signal peptide) and *StDIM/DWF1* (*PGSC0003DMG400011801*) were amplified by PCR with primers listed in S1 Table. The fragments were cloned in TOPO Gateway vector (Invitrogen) and then recombined into binary BiFC-Gateway destination vectors: pDEST–GWVYNE and pDEST–VYNE(R)GW (express the N-terminus of Venus corresponding to aa 1–173 upstream or downstream to the multicloning site, respectively) or pDEST–GWVYCE and pDEST–VYCE(R)GW (express the C-terminal fragment of Venus corresponding to aa 156–239 upstream or downstream to the multicloning site, respectively) [44, 45]. *Agrobacterium tumefaciens* cells (strain GV3101) carrying the different constructs were grown overnight in 10 ml of LB (supplemented with gentamycin, rifampycin and kanamicin). The culture was pelleted and resuspended in 10 mM MES in the presence of 100 μM acetosiryngone (adjusted OD = 0.6) and incubated for 3 h in darkness. Young leaves of 3–4 week-old *Nicotiana benthamiana* plants were co-infiltrated with an equal mix of both cultures. Images were acquired 2–3 days after infiltration under a Leica TCS-SP5 Confocal Microscope (Leica Microsystems, Germany; at Laboratorio Integral de Microscopía, CICVyA, INTA) using a 63X water immersion objective.

### Plant transformation and histochemical localization studies

Potato plants were transformed via GV3101 *Agrobacterium tumefaciens* strain with the PStSN1::GUS construct (Almasia *et al*., 2010) as described by del Vas (1992) [46]. Equivalent constructs without promoter (EV::GUS) or with the *Cauliflower mosaic virus* 35S promoter (35S::GUS) were used as negative and positive controls, respectively.

Plants were histochemically stained for GUS visualization by immersing in 5-bromo-4-chloro-3-indolyl-beta-D-glucuronic acid, cyclohexylammonium salt solution (X-gluc) [47] and incubating for 16 h at 37°C. Chlorophyll was then removed from tissues by immersion in 90% ethanol. Samples were observed and photographed under Nikon SMZ-2T (Japan) magnifying glass or Leica TCS-SP5 Confocal Microscope (Germany) for differential interference contrast imaging.

### Statistical analyses

Statistical differences in ROS, ascorbic acid, and SA and GA content levels between *Snakin-1* silenced lines (A2 and A3) and WT plants were tested using Student’s t-test (InfoStat, http://www.infostat.com.ar). Statistical analysis of relative gene expression ratios were performed by fgStatistics software interface (http://sites.google.com/site/fgStatistics/) using the algorithm developed by Pfaffl and associates [48].

## Results

### *Snakin-1* silencing enhaced ROS and reduced ascorbic acid levels

**Fig 1:**
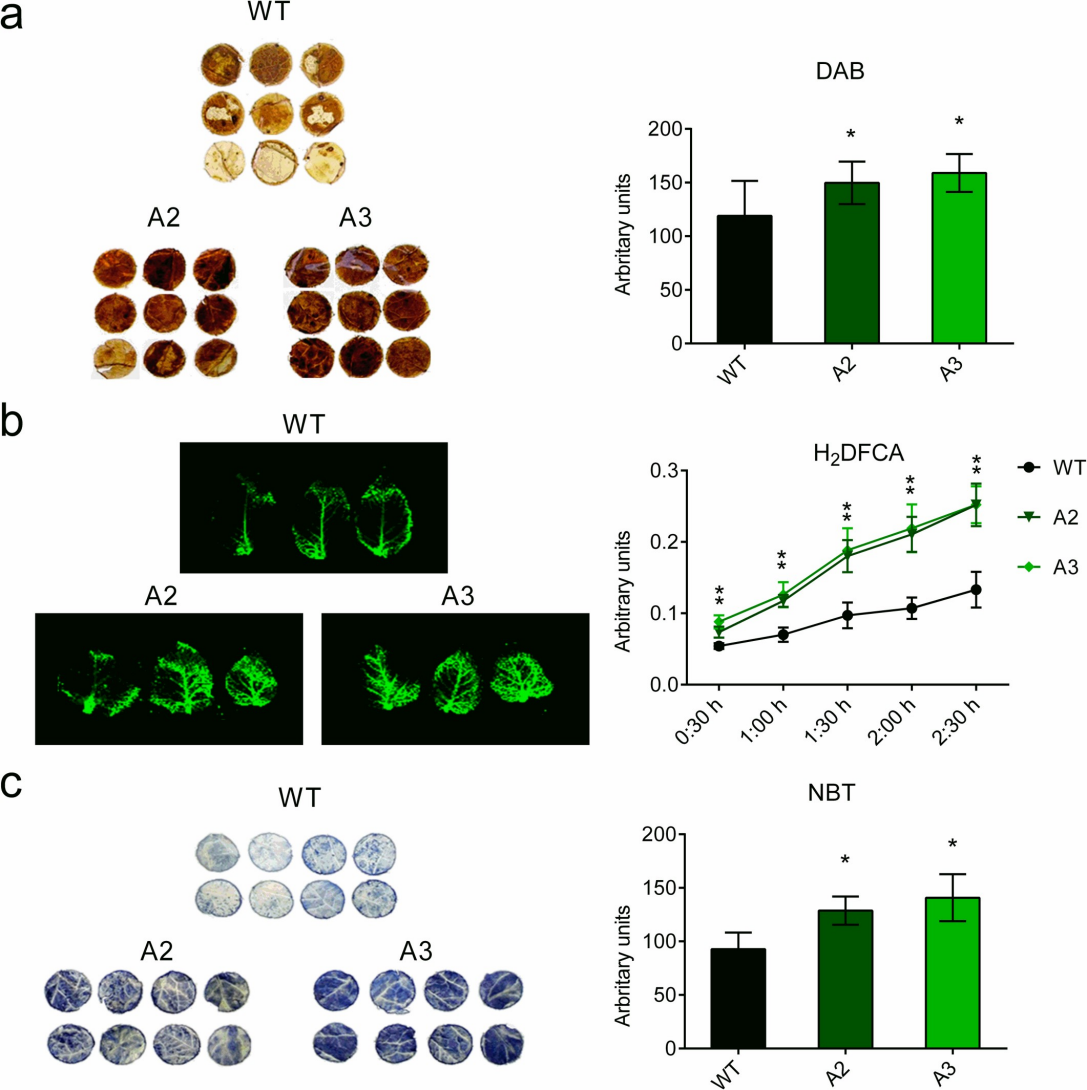
Histochemical staining of leaf tissues from transgenic plants. Potato leaves were stained with (a) DAB and (b) H_2_DCF-DA to detect peroxide level and with (c) NBT staining to detect O_2_^–^. For quantitative studies, images were analyzed with ImageJ. Scale bars represent quantification measured in arbitrary units (means ± SD). For detection of peroxide production over time with H_2_DCF-DA, leaf discs were incubated with the solution and the changes in absorbance (488 nm) were measured by spectrophotometry (quantitative data are means ± SD of fluorescence values). WT: wild type; A2 and A3: *Snakin-1* silenced lines. Asterisks indicate significant differences between transgenic plants compared with the WT (P value < 0.05).

To study whether *Snakin-1* affects the redox balance in potato, we analyzed ROS levels in two independent transgenic *Snakin-1* silenced lines (A2 and A3) and WT plants. Histochemical stainings revealed higher levels of peroxides and O_2_^-^ in A2 and A3 leaves with respect to WT ones (Fig 1). The quantification of DAB and NBT stainings and H_2_DCF-DA absorbance showed a significant increase of ROS in A2 and A3 lines. These results demonstrates that ROS content is altered in the *Snakin-1* silenced lines.

**Fig 2:**
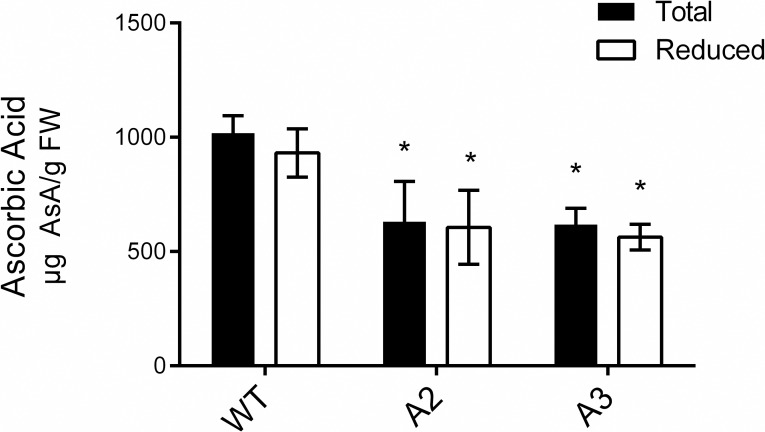
Total (reduced plus oxidized) and reduced ascorbic acid determination by high-performance liquid chromatography. Data are the mean ± standard error of six replicates. Asterisks indicate significant differences between transgenic plants compared with the WT (P value < 0.05).

Because of the altered ROS levelsobserved in *Snakin-1* silenced lines and since ascorbic acid is the most important antioxidant in plants, we assessed the levels of total (reduced plus oxidized) and reduced ascorbic acid through HPLC. In this assay, the fully expanded leaves of A2 and A3 silenced lines displayed significant decreased levels of ascorbic acid content by 40% with respect to WT (Fig 2).

### *Snakin-1* silencing enhanced GA and SA levels

The expression of most of Snakin/GASA genes is modulated by plant hormones and, recently, we have demonstrated that the expression of *Snakin-1* is downregulated by GA [32]. Since the overexpression of *FsGASA4* and *GASA5*, two members of Snakin/GASA family regulated by GA, alters levels and/or responses of SA in Arabidopsis [27, 28], we investigate whether *Snakin-1* is involved in hormone homeostasis.

**Fig 3:**
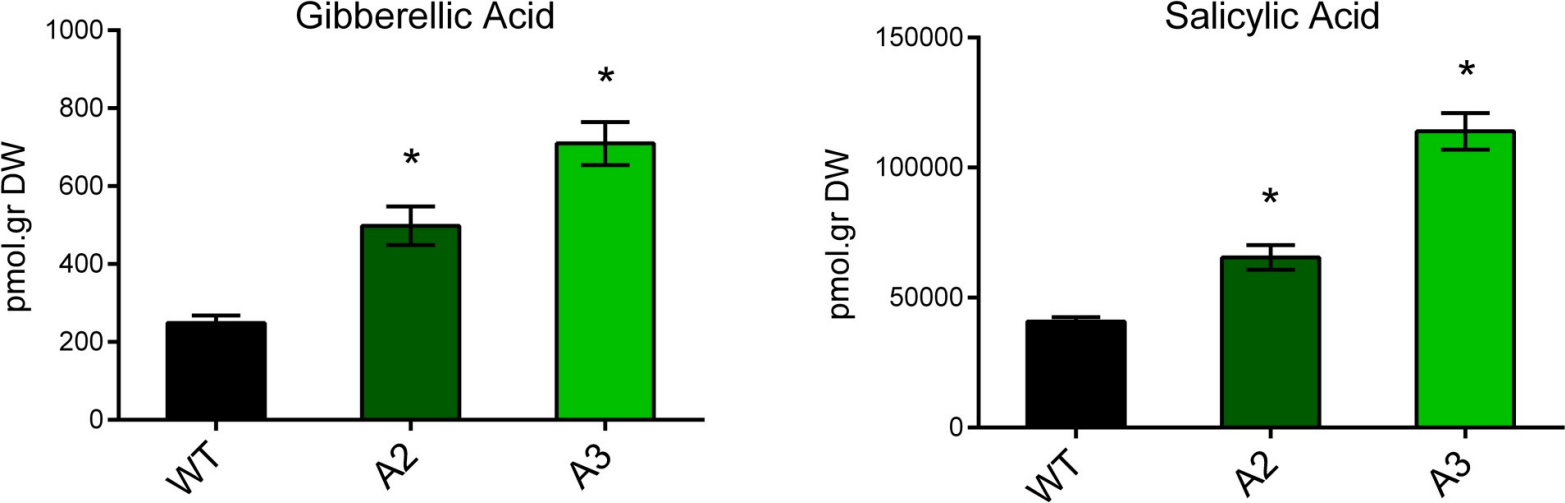
GA and SA levels determined by HPLC in transgenic lines. The mean ± standard error of four replicates is shown. Significant differences from the WT according to Student’s t test are indicated with asterisks (P value < 0.05).

The endogenous levels of two hormones related to stress responses, as well as to plant growth and development were mesasured in leaves of transgenic and WT plants. Leaves of both *Snakin-1* silenced lines (A2 and A3) showed a significant increase of GA_1_ and SA levels with respect to WT (Fig 3).

### Transcriptome analyses of *Snakin-1* silenced lines

In order to gain more insights into the molecular mechanisms underlying the roles of *Snakin-1*, transcriptomes from young leaves samples of A2 silenced line and WT were analysed using RNA-Seq.

**Fig 4:**
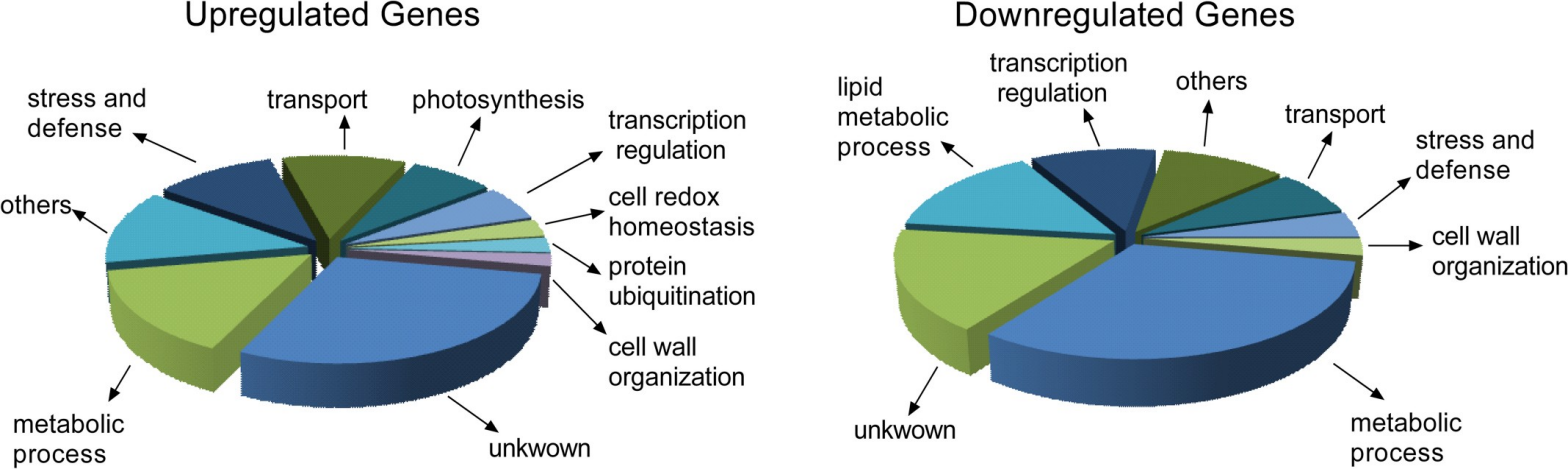
Classification in functional categories of differentially expressed genes. Differentially expressed genes (238 upregulated and 214 downregulated, Q <0.01) were classified according to their functions in categories based on sequence similarity using public databases (Potato Genome Sequencing Consortium database, Blast2GO, KEGG, NCBI, Sol Genomics Network).

Differentially expressed genes (S2 Table, 238 upregulated and 214 downregulated, Q <0.01) were classified according to their functions in categories based on sequence similarity using public databases (Potato Genome Sequencing Consortium database, Blast2GO, KEGG, NCBI, Sol Genomics Network). In the silenced line A2, the highest proportion of the upregulated transcripts with assigned function were grouped in metabolic process, stress and defense responses, transport and photosynthesis. In addition, we also identified genes involved in transcription regulation, cell redox homeostasis, protein ubiquitination and cell wall organization. Most of the downregulated transcripts, on the other hand, were involved in metabolic processes, lipid metabolism and transcription regulation. Finally, among the downregulated genes, minor groups included transcripts related to transport, stress and defense and cell wall organization (Fig 4).

As a complementary approach to determine the signaling and metabolic pathways that are controlled by *Snakin-1*, we next performed an enrichment analysis of the transcriptomic data according to the biological process using PANTHER [49]. This study revealed a significant over-representation of five functional categories among the upregulated genes: sulfur compound metabolic process, response to abiotic stimulus, homeostatic process, cellular amino acid metabolic process and RNA metabolic process. The downregulated genes were significantly enriched in functional categories related to fatty acid metabolic process, steroid metabolic process and lipid metabolic process (Table 1).

**Table 1:** Enrichment analysis of the transcriptomic data according to the biological process using PANTHER (FDR <0.01).

**Functional categories enrichment among the downregulated genes (Q < 0.01)**			
**PANTHER GO-Slim Biological Process**	**Fold Enrichment**	**+/-**	**FDR**
fatty acid metabolic process	7.65	+	4.77E-03
steroid metabolic process	6.23	+	3.76E-03
lipid metabolic process	6.05	+	5.21E-08
**Functional categories enrichment among the upregulated genes (Q < 0.01)**			
**PANTHER GO-Slim Biological Process**	**Fold Enrichment**	**+/-**	**FDR**
sulfur compound metabolic process	6.78	+	2.01E-02
response to abiotic stimulus	5.67	+	3.88E-02
homeostatic process	5.32	+	4.27E-02
cellular amino acid metabolic process	3.50	+	4.60E-02
RNA metabolic process	0.09	-	1.83E-02

**Fig 5:**
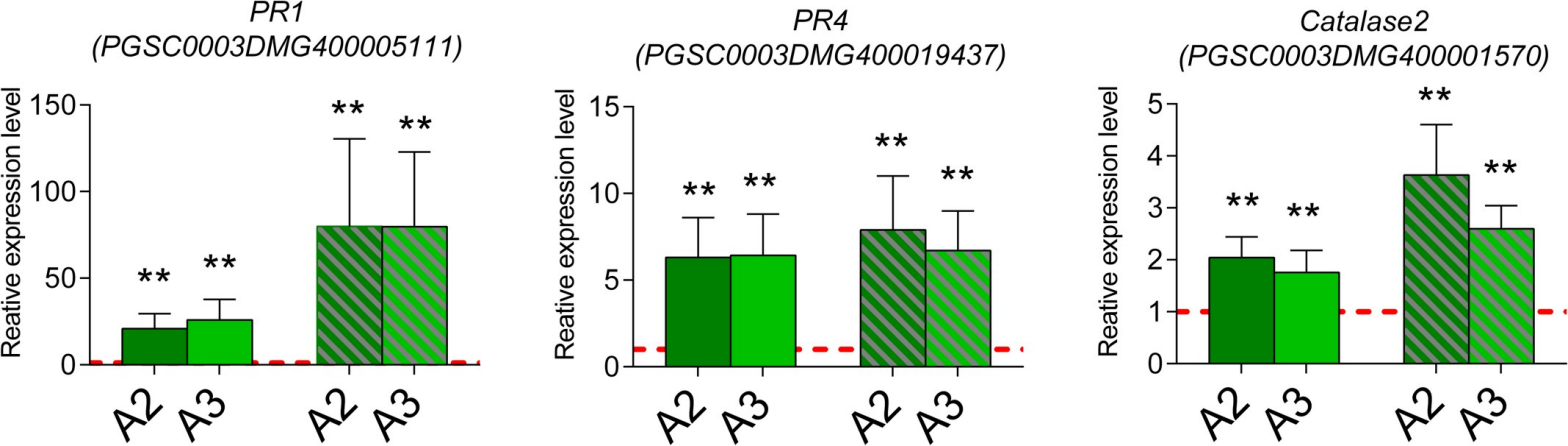
Validation by qRT-PCR of selected genes from RNA sequencing analyses. Upregulated transcripts in *Snakin-1* silenced lines. Dash lines correspond to the expression level of WT plants. Asterisks indicate significant differences (* :  P value < 0.05 and **: P value <0.01). Solid bars: young leaves; striped bars: fully expanded leaves.

Considering our previous results, among the upregulated transcripts in *Snakin-1* silenced line A2, we narrowed down the validation of transcriptomic data to genes involved in SA signaling pathway and redox homeostasis. Expression levels were validated by qRT-PCR and this analysis was extended to both *Snakin-1* silenced lines A2 and A3 (young and fully expanded leaves). The qRT-PCR results were consistent with RNA-Seq data showing that the expression levels of *PR1*, *PR4* and *Catalase2* were significantly enhanced in leaves of both lines (Fig 5, S3 Table).

**Fig 6:**
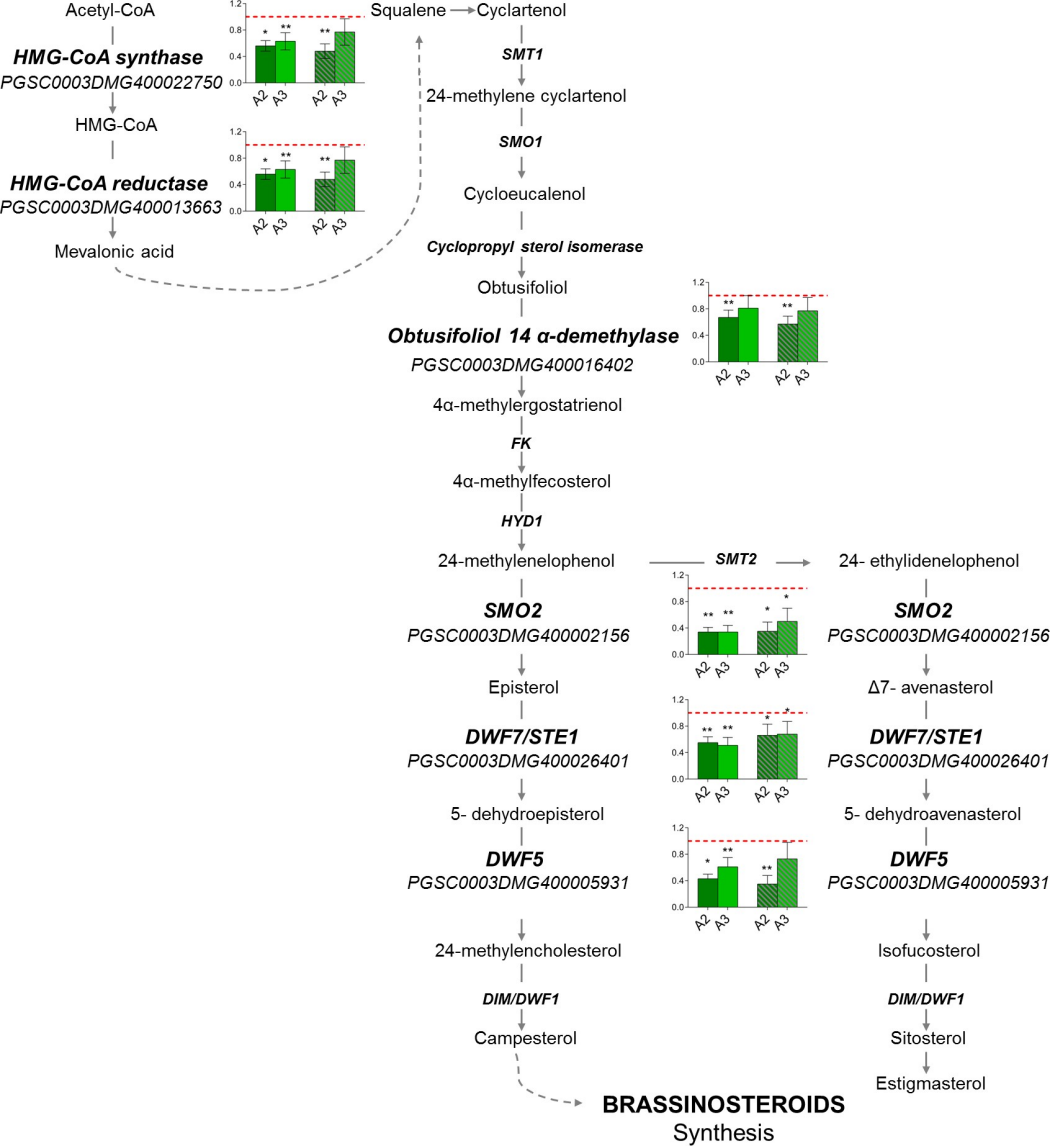
qRT-PCR of genes involved in sterol biosynthesis. Dash lines correspond to the expression level of WT plants. Asterisks indicate significant differences (*: P value < 0.05 and **: P value <0.01). Solid bars: young leaves; striped bars: fully expanded leaves.

Interestingly, many genes related to sterol biosynthesis were downregulated in *Snakin-1* silenced line A2 according to the functional annotation searches and enrichment analyses. The transcriptional changes observed in RNA-Seq analyses were verified by qRT-PCR (Fig 6, S3 Table). The results were in agreement with those obtained by RNA-Seq thus showing that the expression levels of these genes were significantly decreased in leaves of both *Snakin-1* silenced lines (A2 and A3).

### Snakin-1 interacts with StDIM/DWF1

*OsGSR1* RNAi transgenic rice has similar phenotypes to BR-defective mutants and displays a reduced BR level. Moreover, in this study it was demonstrated that OsGSR1 interacts directly with the BR biosynthetic enzyme DIM/ DWF1 [26]. In this context, considering our transcriptomics data and given that *Snakin-1* silencing resulted in phenotypic alterations that resemble BR deficient mutants [31], we next performed BiFC analyses to study whether Snakin-1 is able to interact with the StDIM/DWF1 enzyme.

The complete coding sequence of *Snakin-1* (with its signal peptide, SP) and the sequence encoding the mature peptide (without its signal peptide, ΔSP) were fused to the C-terminal or the N-terminus fragment of Venus in both combinations: as C- or N-terminal protein fusion. Additionally, the coding region of StDIM/DWF1 was fused, at its carboxy-terminus, to the two halves of Venus.

**Fig 7:**
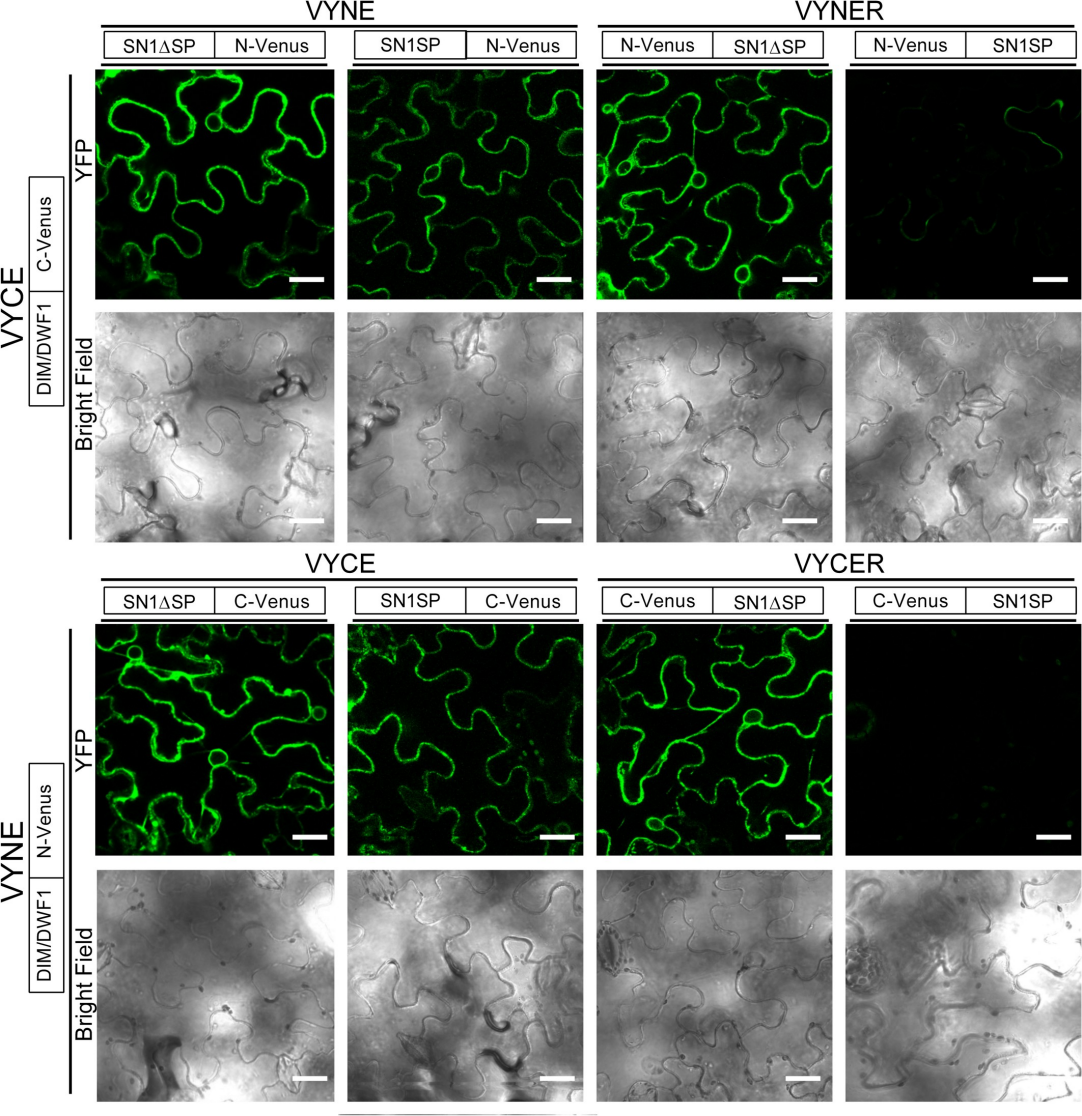
BiFC experiments with transiently expressed Snakin-1 and StDIM/DWF1 fusion proteins. The indicated constructs were co-agroinfiltrated into tobacco leaf epidermal cells. The results are representative of three independent experiments. Scale bar   =   25 μm.

The BiFC assays showed fluorescence signal in *Nicotiana benthamiana* epidermal cells co-expressing Snakin-1 and StDIM/DWF1 in all of the combinations tested, except when the N- or C- terminal of Venus was fused to the full length of Snakin-1 at its N-terminus (Fig 7). In these cases, the presence of any half of Venus fused to 5`extreme of the signal peptide of Snakin-1 may result in conformational changes that hide or alter the interaction sites.

#### Spatial and temporal expression patterns of PStSN1::GUS in potato

**Fig 8:**
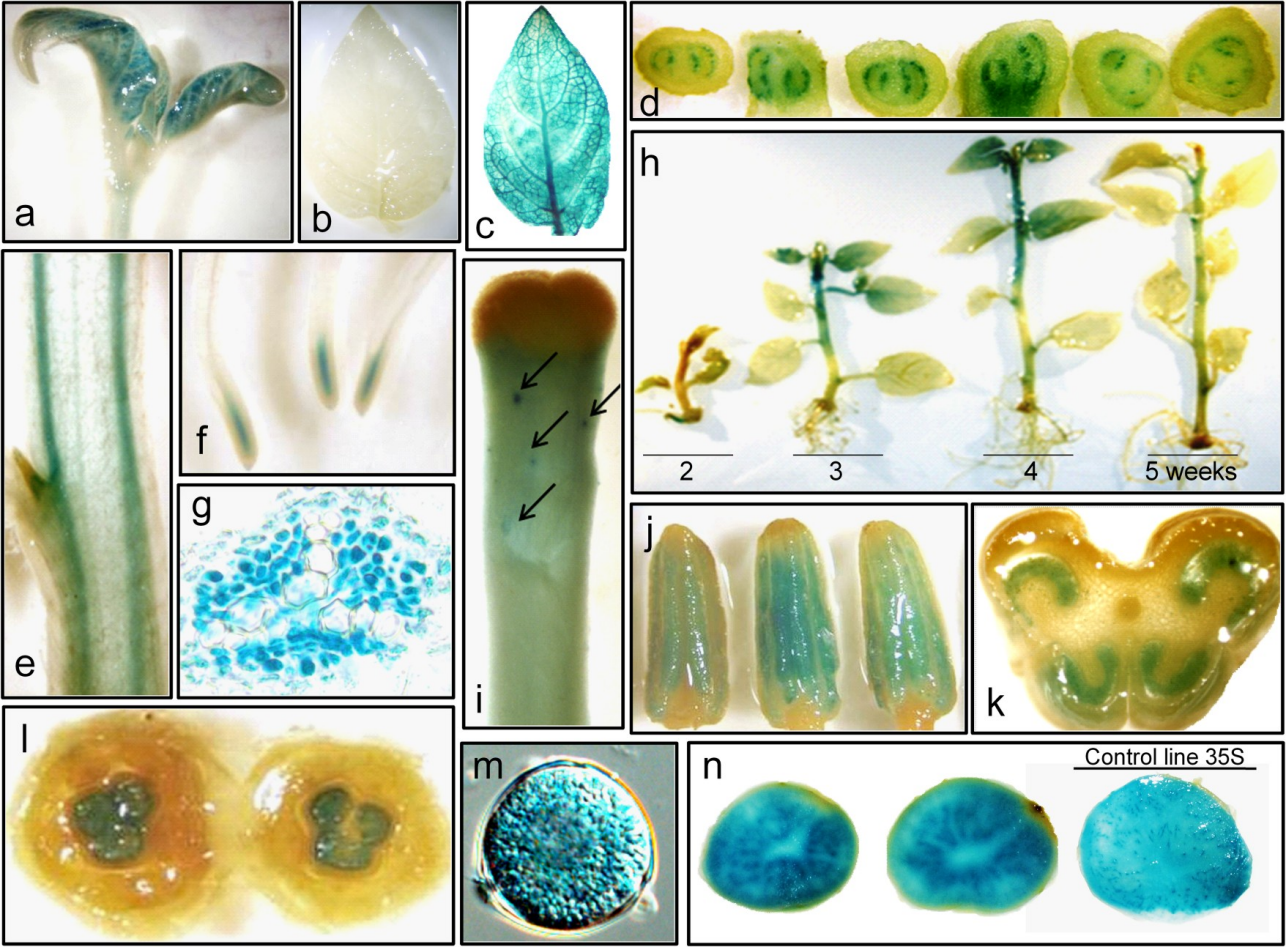
Expression of the reporter protein in PStSN1::GUS transgenic potato plants. (a) Shoot apex. (b) Third leaf of 5-week-old *in vitro* PStSN1::GUS plants (c) Third leaf of 5-week-old *in vitro* 35S::GUS plants. (d) Serial cross-sections of the stem at lateral bud. (e) Longitudinal section of stem. (f) Roots. (g). Detail of root vasculature. (h) Temporal expression: plants were stained over five weeks post micropropagation. (i) Receptacle and floral stigma, arrows stand strong staining areas. (j) Anthers. (k) Cross section of the anther. (l) Cross section of the floral bud and ovary. (m) Microscopic view of a pollen grain. (n) Transverse sections of PStSN1::GUS lines tubers and control 35S line promoter (right). Note the difference of staining in the central pith.

Since the characterization of promoters contributes to the elucidation of the biological function and interactions of the regulated gene, we defined the spatial and temporal expression pattern of a ~1400 bp fragment corresponding to the 5’ upstream region of *Snakin-1* gene (PStSN1) in potato. GUS expression was monitored by histochemical staining of PStSN1::GUS transgenic lines. In plants grown in greenhouse, the reporter protein was detected in the shoot apex, in the apical bud, in the vascular stem and root tissue, in root tip, in receptacle and floral stigma, in anthers (pollen grains) and ovaries (mainly in the tissue surrounding the eggs). No signal was observed in carpels, sepals or petals. In tubers, GUS expression was detected particularly in the perimedulla but not in the outer cortex (Fig 8). Moreover, the temporal expression analysis of *in vitro* grown potato plants showed that the reporter protein started to be detectable from the second week of development and this expression was greater in the third and fourth week but decreased in the fifth (Fig 8H). By contrast, in control plants (line 35S) staining intensity was uniform over time.

## Discussion

### *Snakin-1* is involved in ROS and ascorbic acid accumulation

In this work, we showed that *Snakin-1* silenced lines displayed increased levels of ROS which are important players of the complex redox balance. ROS are produced during normal metabolism and in response to various stimuli and act as signaling molecules with regulatory roles in different physiological processes. In this line, redox reactions of cysteine residues are important molecular mechanisms to convert an oxidant signal into a biological response [50]. Our results suggest that *Snakin-1* plays a role in redox balance. One explanation could be that Snakin-1 presents antioxidant activity through the cysteines of the GASA domain as it was previously demonstrated for some members of Snakin/GASA family from petunia, Arabidopsis and rice [22, 23, 25, 51]. On the other hand, in this work, we confirmed that ascorbic acid, the most abundant non-enzymatic scavenger of ROS, is significantly reduced in A2 and A3 lines. This could explain also, at least in part, the enhanced levels of ROS observed in these lines. However, evidence presented in this work does not allow us to unravel the precise relationship between ROS and ascorbic acid. In agreement with this finding, the silencing of genes involved in ascorbic acid biosynthesis effectively reduces its content in transgenic lines and leads to ROS accumulation [52]. Moreover, in tomato, the reduction of ascorbic acid content results in enhanced ROS levels and in activation of pathogenesis-related (PRs) genes as well as of the antioxidant defense system of plants [53]. Interestingly, our transcriptomic analyses revealed the upregulation of some genes involved in defense response such as *PR1* and *PR4* in *Snakin-1* silenced lines. However, evidence presented in this work does not allow us to unravel the precise relationship between ROS and ascorbic acid. Even though ascorbic acid levels are significantly decreased in A2 and A3, the expression of genes involved in its biosynthesis remained unaltered in *Snakin-1* silenced lines according to our RNAseq analysis which may indicate post-transcriptional regulation of the enzymes involved in its synthesis. It was reported that different components of the ascorbate synthesis pathway are regulated not only transcriptionally but also at the level of translation, protein stability and activity [54]. Remarkably, we demonstrated that the expression of *Catalase2*, involved in detoxifying H_2_O_2_, was significantly upregulated in A2 and A3 lines. The increased expression of *Catalase2* could be a compensatory mechanism for the reduced levels of ascorbic acid, as proposed by Zhang et al. (2013) [53]. Recently, Souza et al (2018) highlighted the complexity of plant redox system which is composed of numerous players that act together to adjust redox metabolism and may be able to compensate one another in order to maintain homeostasis. They proposed that redundancy and complementarity between different components of the redox network enable plants to grow efficiently under both normal and stressed conditions [55]. In this work, in addition to Catalase2, among the upregulated transcripts in *Snakin-1* silenced line we also identified genes involved in cell redox homeostasis such as: Nucleoredoxin, Peroxidases and Thioredoxins (Fig 4, S2 Table).

We have previously demonstrated that *Snakin-1* plays a role in plant growth and development besides having a function in defense since its silencing results in altered cell division, leaf primary metabolism and cell wall components [31]. Interestingly, the analysis of the expression pattern of PStSN1::GUS in potato revealed that it is found mainly in young tissues and zones associated with active growth and cell division. In agreement, ascorbic acid levels are maintained in rapidly growing areas, such as root tips, shoots, and floral organs [56] and in vascular tissue in sections of potato stems and tubers [57]. Moreover, in three potato cultivars, ascorbic acid was significantly higher in the pith than in the outer cortex region [58]. This localization concides with GUS expression directed by PStSN1. Thus, we propose that Snakin-1 and ascorbic acid coexist at a specific time in a specific tissue probably to modulate cell division.

### *Snakin-1* is involved in hormonal balance and affects sterol biosynthesis

In this work, we showed that both GA and SA exhibited enhanced levels in leaves of *Snakin-1* silenced lines with respect to WT. The increased SA levels observed in *Snakin-1* silenced lines, in accordance with an enhanced expression of PRs genes, could be related to the ascorbic acid deficiency and higher ROS content. Indeed, Mukherjee et al. (2010) [52] reported the induction of H_2_O_2_ with a consequent accumulation of SA and expression of *PR*s genes in the ascorbic acid-deficient *Arabidopsis thaliana vtc1-1* mutant. The induction of H_2_O_2_ and SA may allow ascorbic acid-deficient plants to adjust redox homeostasis and consequently to optimize growth and development in response to environmental changes [52].

Interestingly, our results demonstrated that a large number of genes related to sterol biosynthesis were downregulated in *Snakin-1* silenced lines. Sterols are essential eukaryotic membrane components responsible for maintaining membrane integrity and its function and consequently they affect the activity of integral membrane proteins [59]. Moreover, sterols are also precursors of BR, an important class of hormones involved in higher plant growth and development [60]. The characterization of mutants defective in enzymes of sterol biosynthetic pathway showed severely altered sterol composition [61]. In some cases, the reduction in sterols substrates leads to a reduction of BR levels and consequently these mutants show phenotypic traits typical of BR deficient plants [61–65].

Our results indicate that *Snakin-1* silenced lines may exhibit alterations in sterol levels and consequently they could also present lower levels of BR. This would explain, at least in part, why *Snakin-1* silencing resulted in reduced height, short robust stems and dark-green leaves with severe alterations of leaf shape [31]. Interestingly, our BiFC analyses indicated that Snakin-1 interacts with the plant sterols biosynthetic enzyme StDIM/DWF1. In rice, OsGSR1 was proposed to be a positive regulator of BR synthesis through the direct binding and activation of the DIM/DWF1 enzyme. Moreover, *OsGSR1* RNAi lines showed phenotypes similar to plants deficient in BR, with reduced levels of endogenous BR and an elevated content of GA [26]. Similarly, *Snakin-1* silenced lines exhibit phenotypic alterations typical of BR deficient mutants and enhanced GA levels. Interestingly, these lines also present altered flowering and tuberization phenotypes (S1 Fig).

Altogether, these findings support the involvement of Snakin/GASA genes in the complex hormone crosstalk.

In summary, we demonstrated that *Snakin-1* silenced lines showed increased levels of ROS and significantly reduced content of ascorbic acid. We showed that *Snakin-1* silencing enhances GA and SA levels and downregulates the expression of sterol biosynthesis genes. Moreover, Snakin-1 interacts with StDIM/DWF1 possibly reducing BR levels. Additionally, the analysis of the expression pattern of PStSN1::GUS in potato showed that *Snakin-1* is present mainly in young tissues associated with active growth and cell division zones. Consequently, *Snakin-1* could act as integrator of endogenous signals and environmental stimuli and participate in redox and hormone balance to modulate plant development and stress tolerance.

## Supporting information

S1 Table List of the primers used in this study. (XLS)

S2 Table Differentially expressed genes (238 upregulated and 214 downregulated, Q <0.01). (XLS)

S3 Table Fold changes of selected genes as determined by RNA-seq and qRT-PCR. (XLS)

S1 Fig Tuberization and flowering phenotype of WT and *Snakin-1* silenced lines. (TIF)

## Abbreviations

BR: Brassinoesteroid
GA: Gibberellic Acid
ROS: Reactive Oxygen Species
SA: Salicylic Acid
WT: Wild Type
PRs: pathogenesis-related genes

## References

[1] Apel K, Hirt H. Reactive oxygen species: metabolism, oxidative stress, and signal transduction. Annu Rev Plant Biol. 2004;55:373–99. 10.1146/annurev.arplant.55.031903.141701 . 15377225

[2] Mittler R, Vanderauwera S, Suzuki N, Miller G, Tognetti VB, Vandepoele K, et al ROS signaling: the new wave? Trends Plant Sci. 2011;16(6):300–9. 10.1016/j.tplants.2011.03.007 . 21482172

[3] Schippers JH, Foyer CH, van Dongen JT. Redox regulation in shoot growth, SAM maintenance and flowering. Curr Opin Plant Biol. 2016;29:121–8. 10.1016/j.pbi.2015.11.009 . 26799134

[4] Schmidt R, Schippers JH. ROS-mediated redox signaling during cell differentiation in plants. Biochim Biophys Acta. 2015;1850(8):1497–508. 10.1016/j.bbagen.2014.12.020 . 25542301

[5] Singh R, Parihar P, Singh S, Mishra RK, Singh VP, Prasad SM. Reactive oxygen species signaling and stomatal movement: Current updates and future perspectives. Redox Biol. 2017;11:213–8. 10.1016/j.redox.2016.11.006 28012436 PMC5192041

[6] Singh R, Singh S, Parihar P, Mishra RK, Tripathi DK, Singh VP, et al Reactive Oxygen Species (ROS): Beneficial Companions of Plants' Developmental Processes. Front Plant Sci. 2016;7:1299 10.3389/fpls.2016.01299 27729914 PMC5037240

[7] Swanson S, Gilroy S. ROS in plant development. Physiol Plant. 2010;138(4):384–92. 10.1111/j.1399-3054.2009.01313.x . 19947976

[8] Torres MA. ROS in biotic interactions. Physiol Plant. 2010;138(4):414–29. 10.1111/j.1399-3054.2009.01326.x . 20002601

[9] Kocsy G, Tari I, Vankova R, Zechmann B, Gulyas Z, Poor P, et al Redox control of plant growth and development. Plant Sci. 2013;211:77–91. 10.1016/j.plantsci.2013.07.004 . 23987814

[10] Mittler R, Vanderauwera S, Gollery M, Van Breusegem F. Reactive oxygen gene network of plants. Trends Plant Sci. 2004;9(10):490–8. 10.1016/j.tplants.2004.08.009 . 15465684

[11] Smirnoff N. Ascorbic acid: metabolism and functions of a multi-facetted molecule. Curr Opin Plant Biol. 2000;3(3):229–35. . 10837263

[12] Smirnoff N, Wheeler GL. Ascorbic acid in plants: biosynthesis and function. Crit Rev Biochem Mol Biol. 2000;35(4):291–314. 10.1080/10409230008984166 . 11005203

[13] Gallie DR. L-ascorbic Acid: a multifunctional molecule supporting plant growth and development. Scientifica (Cairo). 2013;2013:795964 10.1155/2013/795964 24278786 PMC3820358

[14] Olmos E, Kiddle G, Pellny T, Kumar S, Foyer C. Modulation of plant morphology, root architecture, and cell structure by low vitamin C in Arabidopsis thaliana. J Exp Bot. 2006;57(8):1645–55. Epub 2006/05/25. erl010 [pii] 10.1093/jxb/erl010 . 16720601

[15] Gray WM. Hormonal regulation of plant growth and development. PLoS Biol. 2004;2(9):E311 10.1371/journal.pbio.0020311 15367944 PMC516799

[16] Daviere JM, Achard P. A Pivotal Role of DELLAs in Regulating Multiple Hormone Signals. Molecular plant. 2016;9(1):10–20. 10.1016/j.molp.2015.09.011 . 26415696

[17] Depuydt S, Hardtke CS. Hormone signalling crosstalk in plant growth regulation. Curr Biol. 2011;21(9):R365–73. 10.1016/j.cub.2011.03.013 . 21549959

[18] Vanstraelen M, Benkova E. Hormonal interactions in the regulation of plant development. Annu Rev Cell Dev Biol. 2012;28:463–87. 10.1146/annurev-cellbio-101011-155741 . 22856461

[19] Xia XJ, Zhou YH, Shi K, Zhou J, Foyer CH, Yu JQ. Interplay between reactive oxygen species and hormones in the control of plant development and stress tolerance. J Exp Bot. 2015;66(10):2839–56. 10.1093/jxb/erv089 . 25788732

[20] Nahirñak V, Almasia NI, Hopp HE, Vazquez-Rovere C. Snakin/GASA proteins: involvement in hormone crosstalk and redox homeostasis. Plant Signal Behav. 2012;7(8):1004–8. 10.4161/psb.20813 22836500 PMC3474668

[21] Aubert D, Chevillard M, Dorne AM, Arlaud G, Herzog M. Expression patterns of GASA genes in Arabidopsis thaliana: the GASA4 gene is up-regulated by gibberellins in meristematic regions. Plant Mol Biol. 1998;36(6):871–83. . 9520278 10.1023/a:1005938624418

[22] Wigoda N, Ben-Nissan G, Granot D, Schwartz A, Weiss D. The gibberellin-induced, cysteine-rich protein GIP2 from Petunia hybrida exhibits in planta antioxidant activity. Plant J. 2006;48(5):796–805. 10.1111/j.1365-313X.2006.02917.x . 17076804

[23] Rubinovich L, Weiss D. The Arabidopsis cysteine-rich protein GASA4 promotes GA responses and exhibits redox activity in bacteria and in planta. Plant J. 2010;64(6):1018–27. 10.1111/j.1365-313X.2010.04390.x . 21143681

[24] Sun S, Wang H, Yu H, Zhong C, Zhang X, Peng J, et al GASA14 regulates leaf expansion and abiotic stress resistance by modulating reactive oxygen species accumulation. J Exp Bot. 2013;64(6):1637–47. 10.1093/jxb/ert021 . 23378382

[25] Rubinovich L, Ruthstein S, Weiss D. The Arabidopsis cysteine-rich GASA5 is a redox-active metalloprotein that suppresses gibberellin responses. Molecular plant. 2014;7(1):244–7. 10.1093/mp/sst141 . 24157610

[26] Wang L, Wang Z, Xu Y, Joo SH, Kim SK, Xue Z, et al OsGSR1 is involved in crosstalk between gibberellins and brassinosteroids in rice. Plant J. 2009;57(3):498–510. 10.1111/j.1365-313X.2008.03707.x . 18980660

[27] Alonso-Ramirez A, Rodriguez D, Reyes D, Jimenez JA, Nicolas G, Lopez-Climent M, et al Evidence for a role of gibberellins in salicylic acid-modulated early plant responses to abiotic stress in Arabidopsis seeds. Plant Physiol. 2009;150(3):1335–44. 10.1104/pp.109.139352 19439570 PMC2705047

[28] Zhang S, Wang X. Overexpression of GASA5 increases the sensitivity of Arabidopsis to heat stress. J Plant Physiol. 2011;168(17):2093–101. 10.1016/j.jplph.2011.06.010 . 21835493

[29] Segura A, Moreno M, Madueno F, Molina A, Garcia-Olmedo F. Snakin-1, a peptide from potato that is active against plant pathogens. Molecular plant-microbe interactions: MPMI. 1999;12(1):16–23. 10.1094/MPMI.1999.12.1.16 . 9885189

[30] Almasia NI, Bazzini AA, Hopp HE, Vazquez-Rovere C. Overexpression of snakin-1 gene enhances resistance to Rhizoctonia solani and Erwinia carotovora in transgenic potato plants. Mol Plant Pathol. 2008;9(3):329–38. Epub 2008/08/19. MPP469 [pii] 10.1111/j.1364-3703.2008.00469.x . 18705874 PMC6640289

[31] Nahirñak V, Almasia NI, Fernandez PV, Hopp HE, Estevez JM, Carrari F, et al Potato snakin-1 gene silencing affects cell division, primary metabolism, and cell wall composition. Plant Physiol. 2012;158(1):252–63. 10.1104/pp.111.186544 22080603 PMC3252113

[32] Nahirñak V, Rivarola M, Gonzalez de Urreta M, Paniego N, Hopp HE, Almasia NI, et al Genome-wide Analysis of the Snakin/GASA Gene Family in Solanum tuberosum cv. Kennebec. American Journal of Potato Research. 2016;93(2):172–88.

[33] Thordal-Christensen H, Zhang Z, Wei Y, Collinge DB. Subcellular localization of H2O2 in plants. H2O2 accumulation in papillae and hypersensitive response during the barley-powdery mildew interaction. The Plant Journal. 1997;11(6):1187–94.

[34] Ezaki B, Gardner RC, Ezaki Y, Matsumoto H. Expression of aluminum-induced genes in transgenic arabidopsis plants can ameliorate aluminum stress and/or oxidative stress. Plant Physiol. 2000;122(3):657–65. 10712528 10.1104/pp.122.3.657 PMC58900

[35] Rhee SG, Chang TS, Jeong W, Kang D. Methods for detection and measurement of hydrogen peroxide inside and outside of cells. Mol Cells. 2010;29(6):539–49. 10.1007/s10059-010-0082-3 . 20526816

[36] Wohlgemuth H, Mittelstrass K, Kschieschan S, Bender J, Weigel HJ, Overmeyer K, et al Activation of an oxidative burst is a general feature of sensitive plants exposed to the air pollutant ozone. Plant, Cell and Environment 2002;25:717–26.

[37] Rosello PL, Vigliocco AE, Andrade AM, Riera NV, Calafat M, Molas ML, et al Differential hormonal and gene expression dynamics in two inbred sunflower lines with contrasting dormancy level. Plant physiology and biochemistry: PPB. 2016;102:133–40. 10.1016/j.plaphy.2016.02.021 . 26934102

[38] Conti G, Rodriguez MC, Manacorda CA, Asurmendi S. Transgenic expression of Tobacco mosaic virus capsid and movement proteins modulate plant basal defense and biotic stress responses in Nicotiana tabacum. Molecular plant-microbe interactions: MPMI. 2012;25(10):1370–84. 10.1094/MPMI-03-12-0075-R . 22712510

[39] Trapnell C, Hendrickson DG, Sauvageau M, Goff L, Rinn JL, Pachter L. Differential analysis of gene regulation at transcript resolution with RNA-seq. Nat Biotechnol. 2013;31(1):46–53. 10.1038/nbt.2450 23222703 PMC3869392

[40] Mizrachi E, Hefer CA, Ranik M, Joubert F, Myburg AA. De novo assembled expressed gene catalog of a fast-growing Eucalyptus tree produced by Illumina mRNA-Seq. BMC Genomics. 2010;11:681 10.1186/1471-2164-11-681 21122097 PMC3053591

[41] Mortazavi A, Williams BA, McCue K, Schaeffer L, Wold B. Mapping and quantifying mammalian transcriptomes by RNA-Seq. Nat Methods. 2008;5(7):621–8. 10.1038/nmeth.1226 . 18516045 PMC13303166

[42] Ramakers C, Ruijter JM, Deprez RH, Moorman AF. Assumption-free analysis of quantitative real-time polymerase chain reaction (PCR) data. Neurosci Lett. 2003;339(1):62–6. . 12618301 10.1016/s0304-3940(02)01423-4

[43] Nicot N, Hausman JF, Hoffmann L, Evers D. Housekeeping gene selection for real-time RT-PCR normalization in potato during biotic and abiotic stress. J Exp Bot. 2005;56(421):2907–14. 10.1093/jxb/eri285 . 16188960

[44] Gehl C, Waadt R, Kudla J, Mendel RR, Hansch R. New GATEWAY vectors for high throughput analyses of protein-protein interactions by bimolecular fluorescence complementation. Molecular plant. 2009;2(5):1051–8. 10.1093/mp/ssp040 . 19825679

[45] Ricardi MM, Guaimas FF, Gonzalez RM, Burrieza HP, Lopez-Fernandez MP, Jares-Erijman EA, et al Nuclear import and dimerization of tomato ASR1, a water stress-inducible protein exclusive to plants. PloS one. 2012;7(8):e41008 10.1371/journal.pone.0041008 22899993 PMC3416805

[46] del Vas M. Obtención y caracterización de plantas de interés agropecuario. Dissertation, University of Buenos Aires UBA. 1992.

[47] Jefferson RA, Bevan M, Kavanagh T. The use of the Escherichia coli beta-glucuronidase as a gene fusion marker for studies of gene expression in higher plants. Biochem Soc Trans. 1987;15(1):17–8. Epub 1987/02/01. . 3549385 10.1042/bst0150017

[48] Pfaffl MW, Horgan GW, Dempfle L. Relative expression software tool (REST) for group-wise comparison and statistical analysis of relative expression results in real-time PCR. Nucleic acids research. 2002;30(9):e36 11972351 10.1093/nar/30.9.e36 PMC113859

[49] Mi H, Huang X, Muruganujan A, Tang H, Mills C, Kang D, et al PANTHER version 11: expanded annotation data from Gene Ontology and Reactome pathways, and data analysis tool enhancements. Nucleic acids research. 2017;45(D1):D183–D9. 10.1093/nar/gkw1138 27899595 PMC5210595

[50] Paulsen CE, Carroll KS. Cysteine-mediated redox signaling: chemistry, biology, and tools for discovery. Chem Rev. 2013;113(7):4633–79. 10.1021/cr300163e 23514336 PMC4303468

[51] Lee S, Han S, Kim S. Salt- and ABA-inducible OsGASR1 is Involved in Salt Tolerance. J Plant Biol 2015;58(2):96–101. 10.1007/s12374-014-0497-z

[52] Mukherjee M, Larrimore KE, Ahmed NJ, Bedick TS, Barghouthi NT, Traw MB, et al Ascorbic acid deficiency in arabidopsis induces constitutive priming that is dependent on hydrogen peroxide, salicylic acid, and the NPR1 gene. Molecular plant-microbe interactions: MPMI. 2010;23(3):340–51. 10.1094/MPMI-23-3-0340 . 20121455

[53] Zhang C, Ouyang B, Yang C, Zhang X, Liu H, Zhang Y, et al Reducing AsA leads to leaf lesion and defence response in knock-down of the AsA biosynthetic enzyme GDP-D-mannose pyrophosphorylase gene in tomato plant. PloS one. 2013;8(4):e61987 10.1371/journal.pone.0061987 23626761 PMC3633959

[54] Fenech M, Amaya I, Valpuesta V, Botella MA. Vitamin C Content in Fruits: Biosynthesis and Regulation. Front Plant Sci. 2019;9:2006 Published 2019 Jan 24. 10.3389/fpls.2018.02006 30733729 PMC6353827

[55] Souza PVL, Lima-Melo Y, Carvalho FE, Reichheld JP, Fernie AR, Silveira JAG, and Daloso DM. Function and Compensatory Mechanisms Among the Components of the Chloroplastic Redox Network. Critical Reviews in Plant Sciences. 2018. 10.1080/07352689.2018.1528409

[56] Franceschi VR, Tarlyn NM. L-Ascorbic acid is accumulated in source leaf phloem and transported to sink tissues in plants. Plant Physiol. 2002;130(2):649–56. 10.1104/pp.007062 12376632 PMC166594

[57] Chinoy NJ. On the specificity of the alcoholic, acidic silver nitrate reagent for the histochemical localization of ascorbic acid. Histochemie. 1969;20(2):105–7. . 4901925 10.1007/BF00268703

[58] Munshi CB, Mondy NI. Ascorbic Acid and Protein Content of Potatoes in Relation to Tuber Anatomy. Journal of Food Science. 1989;54(1):220–1. 10.1111/j.1365-2621.1989.tb08607.x

[59] Clouse SD. Arabidopsis mutants reveal multiple roles for sterols in plant development. Plant Cell. 2002;14(9):1995–2000. 10.1105/tpc.140930 12215500 PMC543216

[60] Benveniste P. Biosynthesis and accumulation of sterols. Annu Rev Plant Biol. 2004;55:429–57. 10.1146/annurev.arplant.55.031903.141616 . 15377227

[61] Schaller H. New aspects of sterol biosynthesis in growth and development of higher plants. Plant physiology and biochemistry: PPB. 2004;42(6):465–76. 10.1016/j.plaphy.2004.05.012 . 15246059

[62] Choe S, Dilkes BP, Gregory BD, Ross AS, Yuan H, Noguchi T, et al The Arabidopsis dwarf1 mutant is defective in the conversion of 24-methylenecholesterol to campesterol in brassinosteroid biosynthesis. Plant Physiol. 1999;119(3):897–907. 10069828 10.1104/pp.119.3.897 PMC32104

[63] Choe S, Noguchi T, Fujioka S, Takatsuto S, Tissier CP, Gregory BD, et al The Arabidopsis dwf7/ste1 mutant is defective in the delta7 sterol C-5 desaturation step leading to brassinosteroid biosynthesis. Plant Cell. 1999;11(2):207–21. 9927639 PMC144158

[64] Klahre U, Noguchi T, Fujioka S, Takatsuto S, Yokota T, Nomura T, et al The Arabidopsis DIMINUTO/DWARF1 gene encodes a protein involved in steroid synthesis. Plant Cell. 1998;10(10):1677–90. 9761794 10.1105/tpc.10.10.1677 PMC143945

[65] Schaller H. The role of sterols in plant growth and development. Prog Lipid Res. 2003;42(3):163–75. . 12689617 10.1016/s0163-7827(02)00047-4

