# Supplementary figures and images for: Snakin-1 affects reactive oxygen species and ascorbic acid levels and hormone balance in potato
Source: PLoS One. 2019 Mar 25;14(3):e0214165. doi: 10.1371/journal.pone.0214165 (PMC6433472; doi:10.1371/journal.pone.0214165)

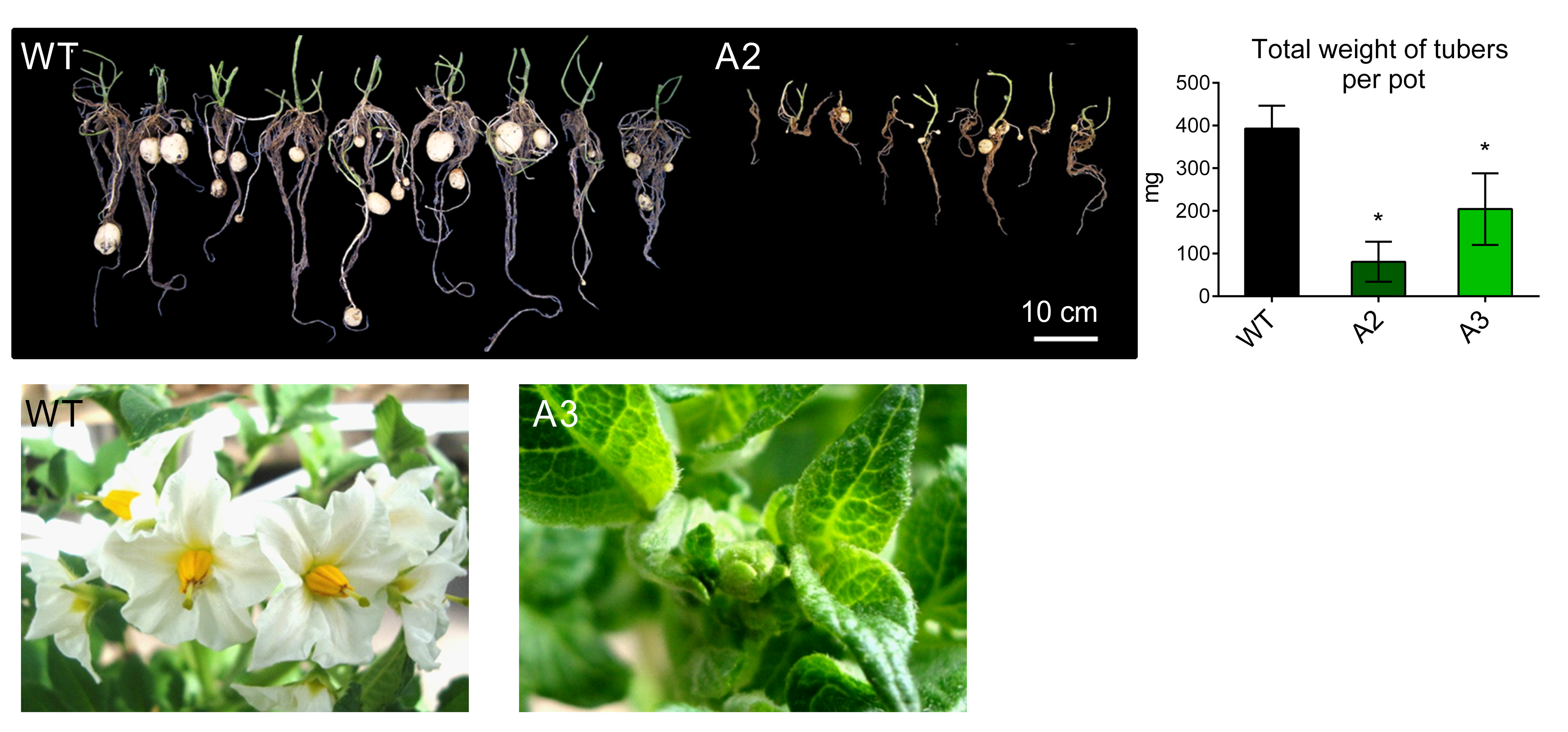

Supplement: S1 Fig — (TIF) [file pone.0214165.s004.tif]
